# 

**Published:** 1915-08

**Authors:** 


### CONTENTS, AUGUST, 1915—VOLUME XXXI, NO. 2.

### ORIGINAL ARTICLES.

The Eradication of Tuberculosis and the Means by Which It May Be Accomplished. By F. C. Smith, M. D. ........ 43
Diarrheal Diseases in Infancy. By Dr. A. Spencer Davis, Dallas, Texas ........ 50

### EUGENICS DEPARTMENT.

Edited by Malone Duggan, San Antonio.

Is War Dsygenic? ........ 55
The Role of the Farmer as an Eugenist ........ 56
War Babies and the Mother ........ 58
A Significant Meeting ........ 59
Rural Morality ........ 61

### EDITORIAL DEPARTMENT.

Medical Revolutions. By T. D. Crothers, M. D., Hartford, Conn.. 64
Justifiable Scope of Medical Journal Advertising. By Dr. G. Henri Bogart, Paris, Ill ........ 66
Pellagra. By Dr. R. H. L. Bibb ........ 68

Abstracts and Selections ........ 71
Items of General Interest ........ 77
Personal News Items ........ 80
Deaths ........ 82
Book Reviews ........ 82
Helpful Hints for the Busy Doctors ........ 84

